# Crop/Weed Discrimination Using a Field Imaging Spectrometer System

**DOI:** 10.3390/s19235154

**Published:** 2019-11-25

**Authors:** Bo Liu, Ru Li, Haidong Li, Guangyong You, Shouguang Yan, Qingxi Tong

**Affiliations:** 1 School of Remote Sensing and Geomatics Engineering, Nanjing University of Information Science and Technology, Nanjing 210044, China; 002700@nuist.edu.cn; 2 Institute of Remote Sensing and Digital Earth, Chinese Academy of Sciences, Beijing 100094, China; liru@radi.ac.cn (R.L.); tongqx@radi.ac.cn (Q.T.); 3 Nanjing Institute of Environmental Sciences, Ministry of Environmental Protection, Nanjing 210042, China; lhd@nies.org (H.L.); ygy@nies.org (G.Y.)

**Keywords:** spectrometer, weed detection, imaging spectroscopy, dimensionality reduction, precision agriculture

## Abstract

Nowadays, sensors begin to play an essential role in smart-agriculture practices. Spectroscopy and the ground-based sensors have inspired widespread interest in the field of weed detection. Most studies focused on detection under ideal conditions, such as indoor or under artificial lighting, and more studies in the actual field environment are needed to test the applicability of this sensor technology. Meanwhile, hyperspectral image data collected by imaging spectrometer often has hundreds of channels and, thus, are large in size and highly redundant in information. Therefore, a key element in this application is to perform dimensionality reduction and feature extraction. However, the processing of highly dimensional spectral imaging data has not been given due attention in recent studies. In this study, a field imaging spectrometer system (FISS; 380–870 nm and 344 bands) was designed and used to discriminate carrot and three weed species (purslane, humifuse, and goosegrass) in the crop field. Dimensionality reduction was performed on the spectral data based on wavelet transform; the wavelet coefficients were extracted and used as the classification features in the weed detection model, and the results were compared with those obtained by using spectral bands as the classification feature. The classification features were selected using Wilks’ statistic-based stepwise selection, and the results of Fisher linear discriminant analysis (LDA) and the highly dimensional data processing-oriented support vector machine (SVM) were compared. The results indicated that multiclass discrimination among weeds or between crops and weeds can be achieved using a limited number of spectral bands (8 bands) with an overall classification accuracy of greater than 85%. When the number of spectral bands increased to 15, the classification accuracy was improved to greater than 90%; further increasing the number of bands did not significantly improve the accuracy. Bands in the red edge region of plant spectra had strong discriminant capability. In terms of classification features, wavelet coefficients outperformed raw spectral bands when there were a limited number of variables. However, the difference between the two was minimal when the number of variables increased to a certain level. Among different discrimination methods, SVM, which is capable of nonlinear classification, performed better.

## 1. Introduction

As awareness of environmental pollution and food safety issues grows, precision agriculture that is efficient, safe, and economical has become the focus of modern agriculture, and site-specific management has become increasingly popular. The traditional method of using herbicide in a large area not only causes environmental pollution but also results in great waste [1]. Traditional methods often perform blanket spaying or use fixed herbicides on entire crop fields instead of areas with particular weeds. Precision weeding using variable spraying and physical weed control has significant advantages in environmental effects, production safety, and economic benefits. The prerequisites for precision weeding are the automatic and rapid discrimination of weeds from crops, which is also an important application and manifestation of site-specific management in agriculture. Numerous studies have tried using various means to recognize weeds and crops, such as machine vision-based methods and spectroscopy, in which the spectral information-based method has received widespread attention for being simple and fast [2,3,4,5,6,7].

Machine vision-based methods have been successfully applied to detect weeds that are distributed between ridges or between sparsely distributed single plants [8]. However, more studies are needed regarding recognizing weeds on ridges, especially when there is no particular pattern in the crop distribution or there are highly variable characteristics in shapes or size at different growth stages (e.g., carrots) [2,7]. The limitations of the machine vision-based method are more evident when individual plants overlap because many crops and weeds are very similar in shapes and colors. Different plant species generally have different spectral characteristics, which change under the impact of factors such as the growth stage, environmental stress, and aging. Many studies have developed spectral imaging systems based on these characteristics and have attempted to use spectroscopy in the detection and discrimination of crops and weeds [9,10,11,12].

Although spectrum-based technology has great application potential in agricultural weed recognition, most studies have focused on indoor conditions or conditions under artificial lighting, where sample preparation or measurement conditions are relatively ideal due to fewer factors that can affect the recognition [2,13,14,15]. The actual field environment is complex and has many factors that can affect the spectral characteristics of plants [15,16]. Can this technology handle and overcome the difficulty caused by the normal, changing natural light condition? This question remains unknown; thus, more studies in the field environment are needed to test the effectiveness of the spectrum-based technology. In addition, hyperspectral imaging data often contain data from approximately hundreds of channels. Although the highly dimensional nature of the data ensures a wealth of information, it also brings an enormous computational load and information redundancy. These challenges are difficult to address using conventional analysis methods. Thus, how to fully utilize effective information in the spectral data and to perform feature extraction and dimensionality reduction on the data are the important prerequisites of the application of imaging spectroscopy. Many studies applied different methods successfully (e.g., principal component analysis, minimum noise fraction, and band selection) to address the problem of dimension reduction especially in hyperspectral remote sensing applications [17,18,19]. These methods can reduce the computational load and information redundancy, thus improving the effectiveness of hyperspectral technology. However, current studies of weed detection have not sufficiently focused on the processing of highly dimensional spectral imaging data.

In this study, a novel field imaging spectrometer system (FISS) (the first FISS in China; now, it upgrades into a series of systems covering different spectral ranges) was used to obtain the spectral data of carrot seedlings and several weed species in the field and to perform the discrimination of crop and weeds. First, the spectral data were normalized (as a preprocessing) and the soil background was removed based on the spectral differences between plants and soils. Then, dimensionality reduction was performed on the spectral data based on the wavelet transform analysis; wavelet coefficients were extracted and used as the features for classification in the weed recognition model, and the classification results were compared with those obtained by using the spectral bands as the classification feature. Finally, a linear discriminant analysis (LDA) model and a support vector machine (SVM) model were used to discriminate weeds from crops. SVM has been widely used in spectral imaging data analysis because of its good performance in highly dimensional data analysis. The objectives of this study can be summarized as follows: (1) to test and examine the application potentials of FISS in crop/weed discrimination under actual field conditions; (2) to select the important spectral bands for the purpose of designing a low-cost multispectral system in crop/weed discrimination; (3) to analyze the impacts of dimensionality reduction of the spectral data and of different classification features (e.g., spectral bands and wavelet coefficients) on the accuracy of crop/weed discrimination; and (4) to compare the capabilities of different discrimination models (e.g., the highly dimensional analysis method of SVM and the conventional LDA method) in discriminating crops and weeds.

## 2. Experimental Design and Data Processing

### 2.1. FISS

**Figure 1:**
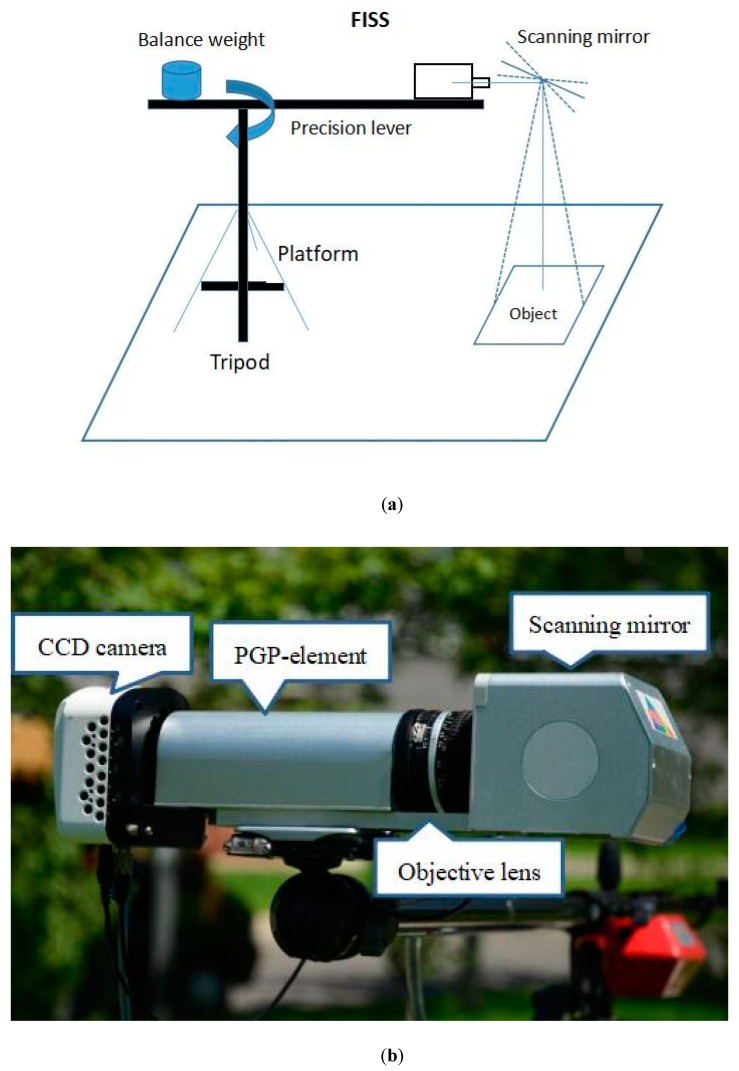
(**a**) Schematic diagram of FISS operation and (**b**) a photograph of the FISS components [16,21].

**Figure 2:**
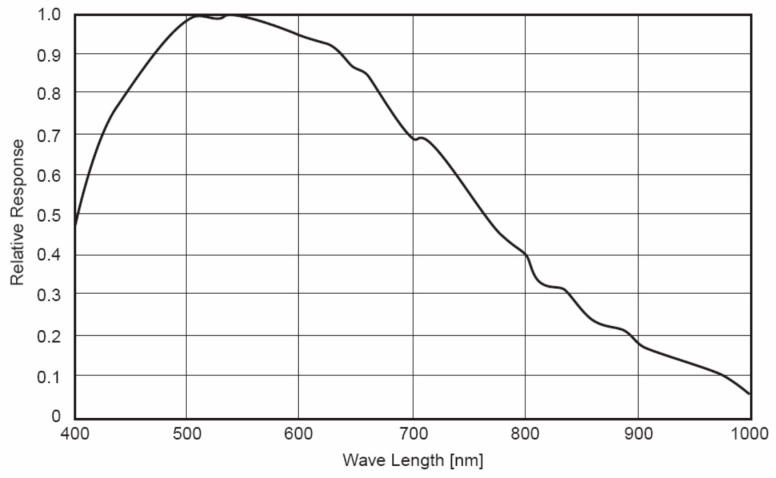
Spectral response of the charge-coupled device (CCD) camera used in FISS.

The FISS consists of three main parts: the computer, optical, and electronic systems (Figure 1). The optical system, which is the key component of the FISS, consists of a scanning mirror, optical lenses, spectroscopic devices (ImSpector V9, Spectral Imaging, Ltd., Finland), and a CCD (charge-coupled device) camera. The CCD resolution is 466 × 344, where 464 denotes the spatial dimensions and 344 denotes the spectral dimensions. Figure 2 shows the spectral response of the CCD camera. The electronic system includes the power and the motor control circuit. The motor control circuit serves primarily to control the rotation of the scanning mirror, to synchronize the beam splitter and receiver, and to collect and store the data.

The hardware is a portable computer used for operating the FISS. The software system includes data collection software and data processing software. The data collection software is used for displaying spectral images and curves and for setting operation parameters such as integral time, aperture, FOV (Field of View), etc. Data processing software is used for data format conversion, geometric correction, radiometric correction, reflectance inversion, etc.

The FISS acquires data by rotating the scanning mirror. The spatial resolution varies with the platform height. The highest resolution is less than 2 mm/pixel. Spatial resolution with platform height is determined the following equation.

(1) r=0.001h

where *r* denotes spatial resolution and *h* denotes platform height. 

Table 1 lists the main technical parameters and performance of the field imaging spectrometer system (FISS) [19,20,21].

### 2.2. Experimental Design

**Figure 3:**
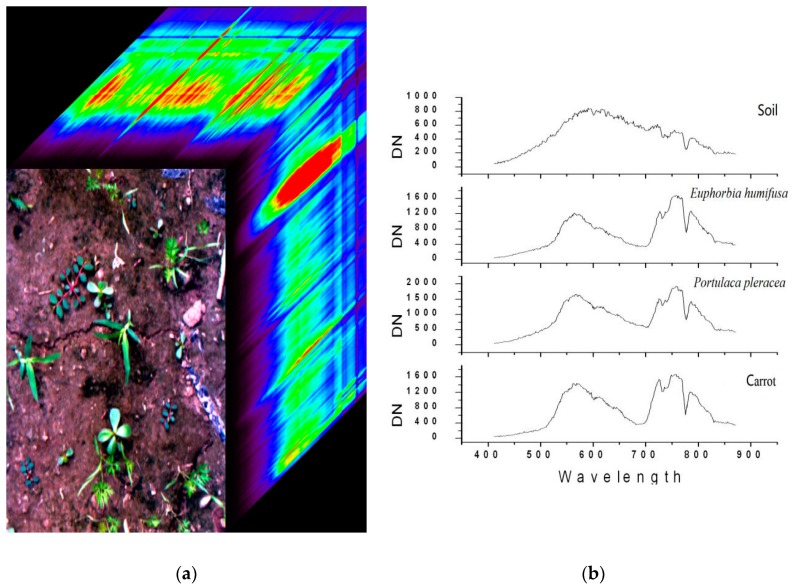
Visual display of spectral imaging data cubes and typical spectra of several surface objects (soil, weeds and crop). (**a**) the sample spectral data that FISS acquired. (**b**) typical spectra of several surface objects.

Hyperspectral data were collected in the farmlands of Wugezhuang village in Beijing on July 30, 2010. The crop (carrot) was planted on July 14, 2010. The date of data collection was the ideal time period for weeding and was the usual time for farmers to manually weed the field. The major weed species were *Portulaca pleracea* (purslane), *Eleusine indica* (goosegrass), and *Euphorbia humifusa* (humifuse), which are widely distributed in the northern region of China. The platform height in the experiment is about 1 m. During the data collection period, the light conditions changed due to the varying cloud cover. The data were collected from 9:30–13:00, and a total of 41 valid images was acquired. This study wants to give an answer to the question on whether this technology can be used successfully under natural light condition, even though the natural light condition definitely could impose some difficulties. Because of this goal, the light condition in this experiment was not strictly consistent and unchanged. The data collection was carefully designed to guarantee that all four plant species are represented in each valid image. Figure 3 shows the sample spectral data that FISS acquired and the typical spectra of several surface objects.

### 2.3. Data Preprocessing

#### 2.3.1. Data Normalization

**Figure 4:**
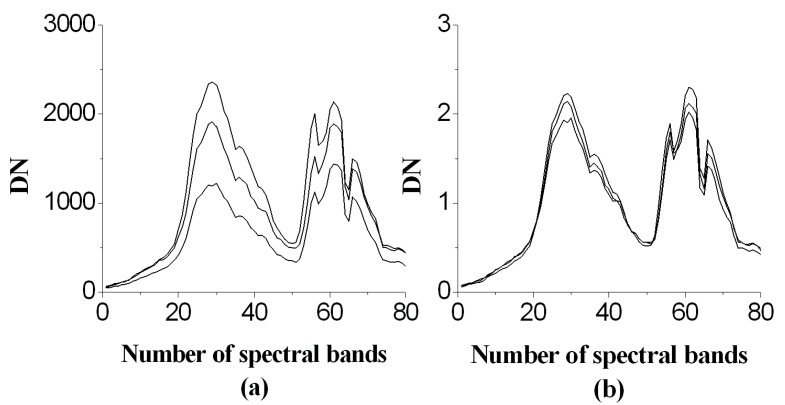
Spectral data normalization: (**a**) raw data; (**b**) normalized data.

Data of wavelengths shorter than 410 nm were discarded due to the low signal-to-noise ratio. To reduce the calculation load and to eliminate noise, the data were averaged across every 4 adjacent bands for the remaining 320 bands, and 80 bands were obtained after averaging. Due to changes in the light conditions during the data collection period, the reflectance of the standard samples could not be measured in real time. The following normalization was conducted on the data to reduce and eliminate the effects of the changes in the light conditions:

(2) $$DN_{i}^{new}=\frac{DN_{i}}{\frac{1}{N}\sum_{k=1}^{N}DN_{k}},$$

where $DN_{i}^{new}$ is the normalized spectral data of the *i*th band, $DN_{i}$ is the digital number (DN) of the spectral data of the *i*th band, and *N* is the number of bands. Figure 4 shows the comparison of the spectra of carrot before and after normalization, indicating that the raw spectra of carrot varied greatly, especially when the weather conditions were not stable. The normalization reduced the difference and, to some extent, reduced the effects of changes in the light conditions.

#### 2.3.2. Soil Background Removal 

**Figure 5:**
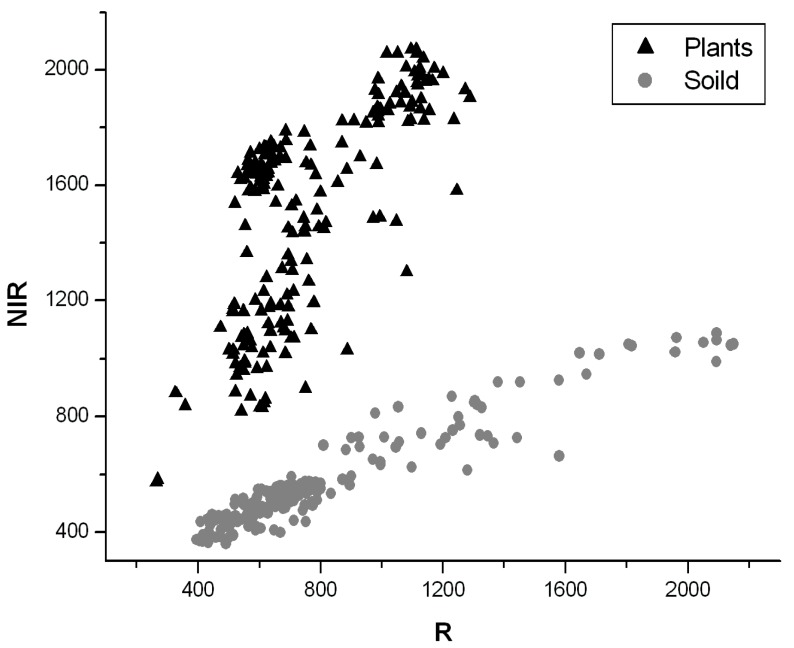
Scatter plot of plants and soil in the near infrared and red bands.

Soil background was removed to speed up data processing. Figure 5 is a scatter plot of randomly selected crops, weeds, and soil in the near infrared and red bands, showing distinct clustering regions for plants and soils. In this study, the NDVI (Normalized Difference Vegetation Index) was used to capture the difference between plants and soil.

(3) $$NDVI=(DN_{\mathrm{NIR}}-DN_{R})/(DN_{\mathrm{NIR}}+DN_{R})$$

where *DN*_NIR_ refers to the DN value of the near infrared band (760 nm) and *DN*_R_ refers to the DN value of the red band (660 nm). Therefore, plants and soils can be distinguished by a threshold calculated based on the histogram of NDVI values. Consequently, the plant pixels can be selected for further analysis.

#### 2.3.3. Sample Collection

Three thousand pixels of training samples and three thousand pixels of validation samples were randomly selected for carrot and for each weed species. To ensure the independence of the data, the training samples and the validation samples were chosen from 20 different images each; thus, a total of 12,000 training samples and 12,000 validation samples were obtained. The training samples were for band selection and model building, and the validation samples were for model validation.

## 3. Crop/Weed Discrimination

Crop/weed discrimination is a typical pattern recognition problem, namely, to train the model based on the spectral difference between crops and weeds. The spectral differences are captured and explored to establish the discrimination model. This process involves feature extraction and discrimination model selection.

### 3.1. Features Used for Classification

Spectral imaging data often contain hundreds of channels; although this highly dimensional nature can provide a wealth of information, it also brings an enormous computational load and challenges to the conventional analysis methods. Therefore, dimensionality reduction has become an important topic in the processing and application of hyperspectral data. Methods for dimensionality reduction of imaging data are divided into two main categories: band selection and band transformation. Band selection method selects a subset from the original set of bands according to certain criteria. Band transformation method extracts new features through the recombination and optimization of bands. In this category, numerous methods have been proposed and applied for dimensionality reduction such as orthogonal subspace projections, principal component analysis (PCA), and minimum noise fraction (MNF) [17,18,19].

#### 3.1.1. Band Selection

Band selection selects a small subset from the original set of bands to be the classification feature [22]. Most studies have used this method for data classification of spectral imaging. Generally, band selection selects bands by comparing the values of different bands according to certain criteria. In terms of classification and recognition, the primary criterion is the importance of a certain band, which is often called the separability. A scatter matrix-based separability was adopted in our study and defined as follows [23]:

(4) $$J=\frac{\left|S_{m}\right|}{\left|S_{w}\right|},$$

where $S_{w}$ is the within-class deviation matrix, $S_{m}$ is the total deviation matrix, and both can be derived based on the statistics of the sample data (the specific calculation method was described in the literature). The band subset can be selected with stepwise selection by comparing the separability (*J*). The stepwise selection constructs a Wilks statistic based on *J* to determine the magnitude of changes in the importance of the remaining variables after introducing or removing variables and, therefore, to determine whether to introduce or remove the variables [24].

#### 3.1.2. Wavelet Transform

Wavelet transform is often used for data compression, noise elimination, and information extraction and has been widely used in digital signal processing, seismic wave analysis, hyperspectral image processing, and other fields [25,26,27,28,29].

The discrete wavelet transform (DWT) of a function, f(λ) (in this case, the pixel spectra of spectral imaging data), can be calculated using the following formula:

(5) $$W_{f\left(\lambda \right)}(j,k)=<f(\lambda ),\varphi_{j,k}(\lambda )>,$$

Its *j*th scale signal energy can be written as follows:

(6) $$E_{j}=\sqrt{\frac{1}{K}\sum_{k=1}^{K}W_{f(\lambda )}(j,k)},$$

where $W_{f\left(\lambda \right)}(j,k)$ is the *k*th coefficient of the level j decomposition, $\varphi_{j,k}(\lambda )$ is the discrete wavelet function, $E_{j}$ is the energy coefficient of the wavelet, and K is the number of coefficients of the level j decomposition. The dimensionality reduction of the hyperspectral data and information extraction can be achieved using wavelet transform to decompose the hyperspectral data. Wavelet transform depends, to a large extent, on the selection of the mother wavelet. Daubechies wavelet function has been found to perform well, and thus, Daubechies wavelet function is used in this study. In our study, wavelet transform was used for the dimensionality reduction of the spectral imaging data and feature extraction and the wavelet coefficients were used as the features for classification. Stepwise regression was used to select a subset of wavelet coefficients with good classification performance.

### 3.2. Discrimination Model 

There are a lot of classification models which could be used for the discrimination task such as spectral angle mapper, minimum distance classification, maximum likelihood classification, artificial neural network, decision tree, and many other machine learning methods [18,19]. In this study, to compare the performance of linear and nonlinear models in crop and weed discrimination, we used the Fisher linear discriminant analysis (LDA) model and the support vector machine (SVM) model; the former is a typical linear simple model, and the latter is a highly complex model and has excellent performance in highly dimensional data processing. The merit of highly dimensional data processing makes it an ideal choice for hyperspectral data processing.

#### 3.2.1. LDA Model 

Fisher’s LDA selects a proper projection axis so that all of the sample data points can be projected onto this axis to generate a value. The requirement for the direction of this projection axis is that the within-class deviation of the projected values should be as small as possible and that the between-class deviation of the projected values should be as large as possible, i.e., maximal difference between between-class means and minimal within-class variance. 

The Fisher criterion is as follows:

(7) $$J(w)=\frac{w^{T}S_{b}w}{w^{T}S_{w}w},$$

where $S_{b}$ is the within-class covariance matrix, $S_{w}$ is the between-class covariance matrix, and w is the weight vector. The specific definition and implementation are described in the literature [23]. Then, the centers of the various classes in the projected space can be calculated with the weight vector; thus, the distance between the sample points and the centers of the various classes in the projected space can be compared and the class to which the unknown sample point belongs can be determined. LDA was implemented using Matlab.

#### 3.2.2. SVM Model 

SVM, first proposed by Vapnik, is a classification algorithm for small samples with minimal separation. SVM can maximize the separation between classes by searching for the hyperplane that can properly separate two-class classification problems [30,31]. SVM uses kernel methods to solve the classification of nonlinear problems. The core idea is to project nonlinear problems in a low-dimensional feature space into a highly dimensional feature space, so that the projected data are linearly separable, simplifying the problem of solving linear SVM classification problems. The small-sample learning capability of SVM fits the needs of hyperspectral data processing and, thus, is widely used in hyperspectral remote sensing (spectral imaging) classification [22,23,24,25,26,27,28,29,30,31,32,33,34].

SVM analysis was implemented using Libsvm in the Python language environment, and the kernel function is a radial basis function. Because SVM depends heavily on the choice of model parameters, we performed grid search optimization on the model parameters to search for optimal parameters (a detailed discussion of SVM occurs in Reference [35]).

## 4. Results and Discussion 

Two types of classification features (spectral bands and wavelet coefficients) and two discrimination models (Fisher LDA model and SVM model) were used in our study for crop and weed identification. Whether spectral bands or wavelet coefficients were used as the classification feature, Wilks’ statistic-based stepwise selection was first used to select variables and, then, LDA and SVM were used for discrimination and classification. Classification accuracy was chosen as the measure of model performance.

### 4.1. Spectral Bands as Classification Features for Crop/Weed Discrimination

**Figure 6:**
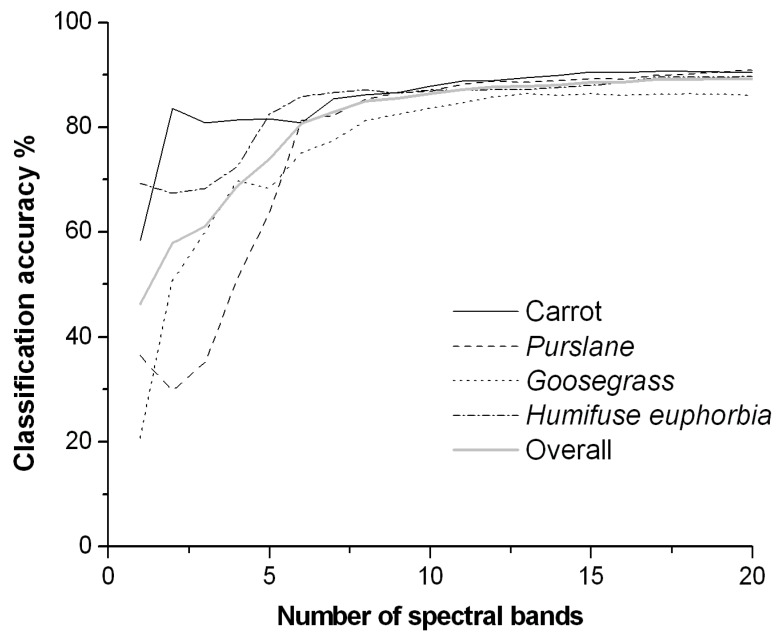
Accuracy of crop/weed multiclass discrimination (linear discriminant analysis (LDA)).

Figure 6 shows the accuracy of crop/weep classification with respect to the number of bands when using the LDA method. The specific data are shown in Table 2. With the increasing number of bands selected, the overall classification accuracy of the three weed species and carrot increase rapidly. In particular, when the number of bands was 8, the recognition rate was 81–88% and, when the number of bands increased to 15, the highest overall classification accuracy was greater than 91%. Any further increase in the number of bands caused only a minimal increase in the classification accuracy, and the accuracy stabilized; even using all 80 bands did not greatly increase the accuracy of the various classes. These results clearly indicated the serious information redundancy of spectral imaging data. Therefore, using the first eight bands selected can achieve satisfactory results in the discrimination of carrot and three weed species. The central wavelengths of the eight bands were, sequentially, 585, 714, 608, 732, 434, 827, 696, and 661 nm. In particular, the 714, 732, and 696 nm bands are located in the red edge region of plants spectra. These three bands primarily reflect the difference in the leaf structures of plants and are an important diagnostic feature for green plants, which is consistent with the vegetation remote sensing theory [22].

Figure 6 shows that purslane and goosegrass were difficult to recognize, whereas carrot and humifuse were easier to discriminate, with classification accuracy up to 80% using only four bands. When the number of bands was less than 5, instead of simply increasing with the increasing number of bands, the classification accuracy of some classes actually fluctuated. This fluctuation is because the bands were selected based on the overall separability of all classes, and thus, the detection accuracy of every class does not necessarily increase with increasing numbers of bands; as shown in the figure, the fact that the overall classification accuracy gradually increased with increasing numbers of bands is a good illustration of this phenomenon.

### 4.2. Wavelet Coefficients as Classification Features in Crop/Weed Discrimination 

When using the wavelet coefficients as the classification feature, Daubechies mother wavelet with several support lengths were selected. The results showed that the classification accuracy of the db3 wavelet was better when n = 3; however, the difference was minimal. Therefore, only the analysis using db3 is presented here. Discrete wavelet transform (DWT) was performed on the 80-dimensional spectral data using db3 as the mother wavelet, and 111 wavelet coefficients were obtained.

Table 3 presents the results using wavelet coefficients as the classification feature, showing that the accuracy changed in a similar trend to that seen when using the raw spectral bands as the classification feature. The accuracy of various classes was greater than or equal to 80% when 8 coefficients were used, and the overall classification accuracy was up to 86.7%.

**Figure 7:**
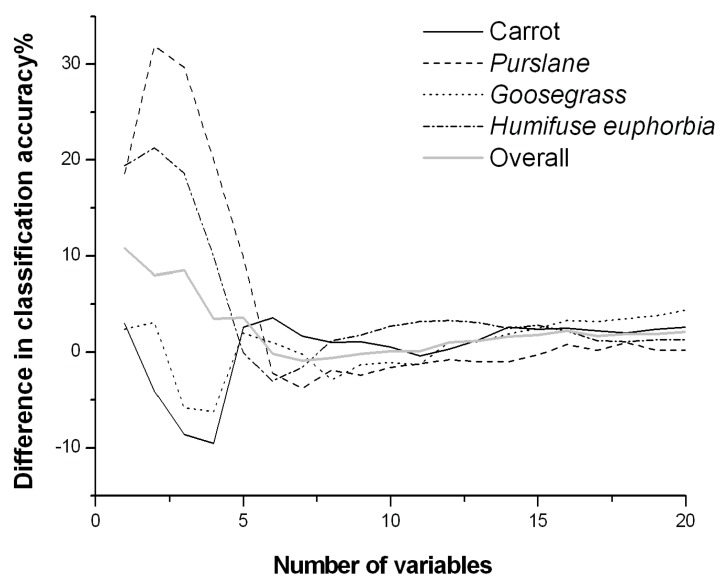
Differences in classification accuracy (CA_WC_-CA_SB_): CA_WC_ is the accuracy of the model using wavelet coefficients as the feature for classification; CA_SB_ is the accuracy of the model using spectral bands as the feature for classification.

To compare the impacts of the spectral bands and wavelet coefficients on the classification accuracy, the difference in classification accuracy between the two given the same number of variables is presented in Figure 7. When fewer variables were selected (1–5), the weed classification accuracy was higher for the wavelet coefficient method than for the spectrum bands. Especially in cases with only 1–2 variables, the accuracy was much higher using wavelet coefficients than using spectral bands (except for carrot) and the overall classification accuracy was greater by 8–10%; with more variables, the accuracy difference between the two gradually decreased until there was essentially no difference. The differences between the two indicated that the wavelet coefficients carried more information when only a few variables were used, and thus, most types of plants were accurately distinguished. Through the scaling transformation and translation of window functions, wavelet analysis is more capable of capturing the local features of the signal and can retain the energy of the low-resolution signals as much as possible at low frequencies. The detailed components of the high-resolution signals contain mainly high-frequency information and noise. Therefore, a single wavelet coefficient variable usually contains more information than a single spectral band. Due to the information redundancy in spectral imaging data, after the number of variables reach a certain number (most of the information in the spectral imaging data is stored in these variables), the effective information carried by either raw bands or wavelet coefficients will no longer increase or will only increase slightly with further increase in the number of variables. Therefore, the classification accuracy of the two tended to stabilize at this time. 

### 4.3. Comparison of Different Discrimination Models (LDA vs. SVM)

**Figure 8:**
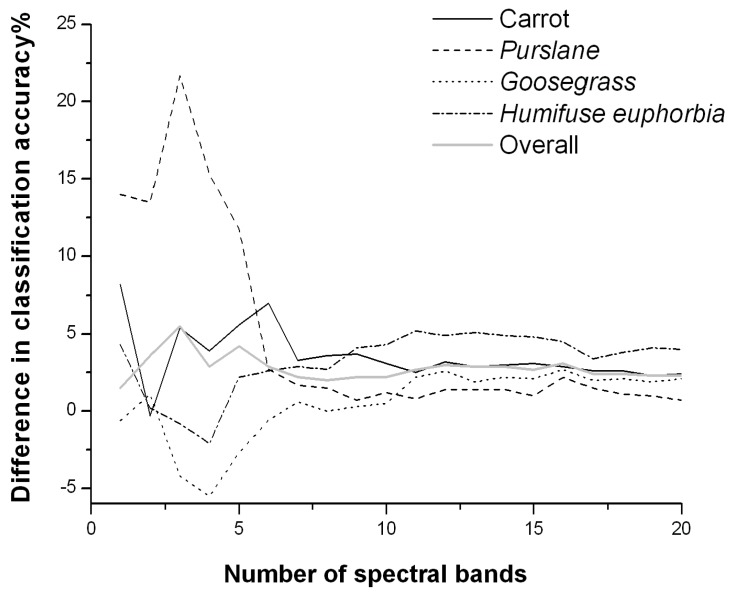
Differences in classification accuracy between the different classification methods (CA_SVM_-CA_LDA_). CA_SVM_ is the accuracy of the SVM model using spectral bands as the feature for classification; CA_LDA_ is the accuracy of the LDA model using spectral bands as the feature for classification.

The comparisons of crop/weed multiclass classification accuracy between LDA and SVM for different combinations of bands are given in Table 2 and Table 3. In all but very few cases, the classification accuracy for various plant species and the overall classification accuracy under different band combinations were generally 1.5–5.5% higher using SVM than using LDA, indicating that a nonlinear classification model is more suitable for crop/weed discrimination and, thus, that the more complex SVM method performs better in classification (Figure 8).

In particular, when the number of bands was greater than 7, the classification accuracy using SVM was better than using LDA in all cases. These results are in accordance with the advantages of SVM, namely, that SVM is more suitable for handling highly dimensional data. Although SVM outperformed LDA in this study, the increase in classification accuracy was not very significant, which might be because a simple model and a small number of classification variables were competent for the task due to the limited number of weed species or the significant spectral difference between weeds and carrot. If the number of weed species increases, SVM is expected to show greater advantages in processing small-sample-size, highly dimensional data. However, this speculation needs to be validated with further studies.

### 4.4. Important Spectral Bands for Multispectral Systems Designing

Because hyperspectral imagers are expensive, it is desirable to achieve satisfactory discrimination results with fewer bands so that lower cost instruments can be designed and developed. For example, a 2-band or 3-band system can be easily achieved using filter technology. Important bands in crop/weed discrimination are shown in Table 4. The classification accuracies of the three bands (Red, Green, Blue) in common digital cameras are also given in the table to illustrate the effectiveness of spectroscopy (only the LDA results are shown in the table).

The best two bands that had the greatest discrimination power in this study were 585 nm and 714 nm. The band 585 nm is located in the spectral region controlled mainly by leaf pigments. It showed that the pigments of these plant leaves played an important role in discriminating weed and crop in our case. The band of 714 nm is located in the spectral region of the “red edge” of vegetation. The abrupt change of reflectance in the “Red edge” region is caused by the combined effects of strong chlorophyll absorption and leaf internal scattering. The reflectance around the red edge is sensitive to a wide range of vegetation chlorophyll content, N content, leaf area index, biomass, and other plant parameters. The red edge is considered to be effective in estimating plant parameters, in assessing plant stress, and in discriminating plant classes [20,36,37]. Our study illustrated that the red edge was an important spectral feature for discriminating crop and weeds. Selected band combinations had good performances because they captured and utilized important spectral features of plants.

In our study, band combinations of 585 and 714 nm or 585, 608, and 714 nm can be used to design low-cost instruments. Interestingly, the classification accuracies of all of the characteristic bands selected were far better than that of conventional RGB bands (an overall accuracy of 77.3%). Indeed, the classification accuracy of a single optimal band (714 nm) (83.6%) was already much higher than that of the 3 bands of RGB, fully demonstrating the large difference in the capability of different bands at weed discrimination as well as the advantage of multispectral/hyperspectral analysis.

## 5. Conclusions

A field imaging spectrometer system (FISS) was used to classify weeds and crops in the natural environment. The raw spectral bands and wavelet coefficients were used as classification features, and Wilks’ statistics-based stepwise selection was used to select bands; the results of two methods, the Fisher LDA and SVM, were then compared.

(1) Multiclass discrimination between weeds and crops or between weeds can be achieved by using only a small number of spectral bands (8 bands), with an overall classification accuracy of greater than 85%; bands in the red edge region played an important role in weeds/crop discrimination, and the overall classification accuracy increased with the increasing number of bands used. The accuracy increased slightly beyond the inflection point and stabilized.
(2) In consideration of the cost of practical application, a 2- or 3-spectral channel instrument with bands 585 and 714 nm or 585, 608, and 714 nm can be considered sufficient for weed detection. This information can be adopted for designing a low-cost multiband system in weed/crop discrimination.
(3) In terms of classification features, wavelet coefficients outperformed raw bands when there were a small number of variables. However, the difference between the two was minimal when the number of variables increased to a certain level. For different discrimination methods, SVM, which is capable of nonlinear classification, performed better. However, the classification accuracy did not improve significantly, which may be due to the limited number of weed species or to significant spectral differences between weeds and carrot, and thus, simple models and a small number of variables were competent for the task. If the number of weed species increases, SVM is expected to show greater advantages in processing small-sample-size, highly dimensional data because of its nonlinear characteristics. However, this speculation needs to be validated with further studies.
(4) Although this preliminary study of weed detection was performed in the field environment and good results were achieved to verify the good performance of FISS, not all factors were comprehensively considered due to the constraints of the experimental conditions. Further studies are needed in the future to investigate the performance of the system under different weather conditions, at different stages of crop seedling, or under conditions with more weed and crop species and to further verify and improve the model. Some issues about the algorithm (e.g., the influence of soil background removal on computation load and the influence of different model training strategies on classification accuracy) need to be well explored and discussed as well.

## Figures and Tables

**Table 1:** Main parameters of the FISS.

Band Number	344	Imaging Rate/(lines/s)	20
Spectral range	379–870 nm	Scan field/°	−20–20
Spectral resolution	4–7 nm	Quantitative value/bit	12
Spatial resolution	The maximum is better than 2 mm	Signal to noise	>500 (60% of bands) *
Radiance calibration precision in laboratory	Better than 5%	Spectral sampling interval/nm	About 1.4

* An integral sphere was used to guarantee that the incident energy is uniform in the Field of View (FOV) of FISS. The signal-to-noise ratio was derived from the calculation of digital number (DN) mean value and root-mean-square value of each band.

**Table 2:** Accuracy of crop/weed classification (using spectral bands as the classification feature).

	Carrot		Purslane		Goosegrass		Humifuse		Overall	
Number of bands	LDA	SVM	LDA	SVM	LDA	SVM	LDA	SVM	LDA	SVM
1	58.5	66.7	36.5	50.5	20.8	20.2	69.3	73.6	46.3	47.8
2	73.6	73.3	29.8	43.3	50.7	51.8	67.4	67.6	57.9	61.5
3	80.9	86.3	35.2	56.9	60.0	55.8	68.3	67.5	61.1	66.6
4	81.4	85.3	51.0	66.3	69.8	64.3	72.5	70.4	68.7	71.6
5	81.6	87.2	63.7	75.5	68.3	65.6	82.5	84.7	74.0	78.2
6	80.9	87.9	81.3	84.0	75.1	74.5	85.9	88.5	80.8	83.7
7	85.4	88.7	82.3	84.0	77.5	78.1	86.6	89.5	82.9	85.1
8	86.2	89.8	85.4	86.9	81.3	81.3	87.2	89.9	85.0	87.0
9	86.7	90.4	86.6	87.3	82.5	82.8	86.5	90.6	85.6	87.8
10	87.8	90.9	86.9	88.1	83.6	84.1	87.2	91.5	86.4	88.6
11	88.8	91.3	88.2	89.0	84.7	86.9	87.0	92.2	87.2	89.9
12	89.0	92.2	88.8	90.2	85.8	88.4	87.2	92.1	87.7	90.7
13	89.5	92.4	88.6	90.0	86.4	88.3	87.2	92.3	87.9	90.8
14	89.9	92.9	88.9	90.3	86.1	88.3	87.6	92.5	88.1	91.0
15	90.6	93.7	89.3	90.3	86.4	88.5	88.0	92.8	88.6	91.3
16	90.5	93.4	89.2	91.4	86.1	88.8	88.5	93.0	88.6	91.7
17	90.7	93.3	89.9	91.4	86.3	88.3	89.6	93.0	89.1	91.5
18	90.7	93.3	90.1	91.2	86.4	88.5	89.6	93.4	89.2	91.6
19	90.6	92.9	90.6	91.6	86.3	88.2	89.6	93.7	89.3	91.6
20	90.5	92.9	90.9	91.6	86.1	88.2	89.7	93.7	89.3	91.6
80	93.7	95.1	92.1	94.0	91.4	93.6	91.3	96.1	92.1	94.7

**Table 3:** Crop/weed classification accuracy (using the wavelet coefficient as the classification feature).

Number of Wavelet Coefficients	Carrot		Purslane		Goosegrass		Humifuse		Overall	
	LDA	SVM	LDA	SVM	LDA	SVM	LDA	SVM	LDA	SVM
1	61.5	71.9	55.1	73.5	23.2	20.3	88.7	87.0	57.1	58.1
2	69.5	67.8	61.7	66.2	53.8	54.9	88.7	87.2	65.9	66.5
3	72.3	78.6	64.9	64.9	54.2	52.9	86.9	86.8	69.6	70.8
4	71.9	79.7	71.0	75.0	63.6	64.1	82.4	87.2	72.2	76.5
5	84.2	87.9	73.6	80.2	70.3	69.3	82.4	87.5	77.6	81.2
6	84.5	90.5	79.1	83.4	76.1	73.4	82.9	89.3	80.6	84.2
7	87.1	91.0	78.5	85.3	77.3	76.4	85.0	91.3	82.0	86.0
8	87.2	90.5	83.5	86.4	78.4	78.4	88.4	92.6	84.4	86.7
9	87.8	91.5	84.2	87.8	81.2	82.2	88.3	93.4	85.4	88.7
10	88.3	91.3	85.3	87.7	82.5	85.4	89.9	93.7	86.5	89.5
11	88.4	92.4	87.0	88.7	83.4	86.3	90.2	94.7	87.3	90.5
12	89.3	93.2	88.0	90.2	86.9	88.6	90.5	95.2	88.7	91.8
13	90.8	93.7	87.6	90.4	87.5	88.8	90.3	94.6	89.1	91.9
14	92.5	94.9	87.9	91.8	88.0	90.0	90.1	95.3	89.7	93.0
15	93.0	94.5	89.0	91.6	88.9	90.8	90.8	94.4	90.4	92.8
16	93.0	95.2	90.0	92.7	89.4	90.9	90.7	95.2	90.8	93.5
17	92.9	95.4	90.1	92.8	89.5	91.3	90.8	95.1	90.8	93.6
18	92.7	95.2	91.1	93.9	89.9	91.3	90.7	95.1	91.1	93.9
19	93.0	95.2	90.8	93.8	90.1	92.0	90.9	95.1	91.2	94.0
20	93.1	95.0	91.1	94.4	90.5	91.5	91.0	95.5	91.4	94.1
111	93.6	94.5	92.3	94.1	91.2	91.6	91.2	94.6	92.1	93.7

**Table 4:** Important band combinations and their respective classification accuracies.

Band Combination (nm)		Carrot Classification Accuracy (%)	Weed Classification Accuracy (%)	Overall Classification Accuracy (%)
585, 714		89.3	81.5	85.4
585, 608, 714		87.3	85.7	86.5
585, 608, 714, 732		90.0	87.3	88.6
434, 585, 608, 714, 732		91.4	87.1	89.3
RGB *	450, 550, 638	79.1	75.6	77.3

* RGB refers to red, green and blue bands.
